# Factors affecting intake, metabolism and health benefits of phenolic acids: do we understand individual variability?

**DOI:** 10.1007/s00394-019-01987-6

**Published:** 2019-05-21

**Authors:** Andreia Bento-Silva, Ville M. Koistinen, Pedro Mena, Maria R. Bronze, Kati Hanhineva, Stefan Sahlstrøm, Vaida Kitrytė, Sofia Moco, Anna-Marja Aura

**Affiliations:** 1 Instituto de Tecnologia Química e Biológica, Universidade Nova de Lisboa (ITQB NOVA), Oeiras, Portugal; 2 Faculty of Pharmacy, University of Lisbon, Lisbon, Portugal; 3 Department of Clinical Nutrition, University of Eastern Finland, Kuopio, Finland; 4 Human Nutrition Unit, Department of Food and Drugs, University of Parma, Parma, Italy; 5 Instituto de Biologia Experimental Tecnológica (iBET), Oeiras, Portugal; 6 Nofima Norwegian Institute of Food Fisheries and Aquaculture, Ås, Norway; 7 Kaunas University of Technology, Kaunas, Lithuania; 8 Nestlé Institute of Health Sciences, Nestlé Research, Lausanne, Switzerland; 9 VTT Technical Research Centre of Finland Ltd, P.O. Box 1000, Tietotie 2, 02044 VTT Espoo, Finland

**Keywords:** Phenolic acids, Colonic metabolites, Bioavailability, Bioactivity, Interindividual variability

## Abstract

**Introduction:**

Phenolic acids are important phenolic compounds widespread in foods, contributing to nutritional and organoleptic properties.

**Factors affceting individual variability:**

The bioavailability of these compounds depends on their free or conjugated presence in food matrices, which is also affected by food processing. Phenolic acids undergo metabolism by the host and residing intestinal microbiota, which causes conjugations and structural modifications of the compounds. Human responses, metabolite profiles and health responses of phenolics, show considerable individual variation, which is affected by absorption, metabolism and genetic variations of subjects.

**Opinion:**

A better understanding of the gut-host interplay and microbiome biochemistry is becoming highly relevant in understanding the impact of diet and its constituents. It is common to study metabolism and health benefits separately, with some exceptions; however, it should be preferred that health responders and non-responders are studied in combination with explanatory metabolite profiles and gene variants. This approach could turn interindividual variation from a problem in human research to an asset for research on personalized nutrition.

## Sources, intake and bioavailability of phenolic acids

**Fig. 1:**
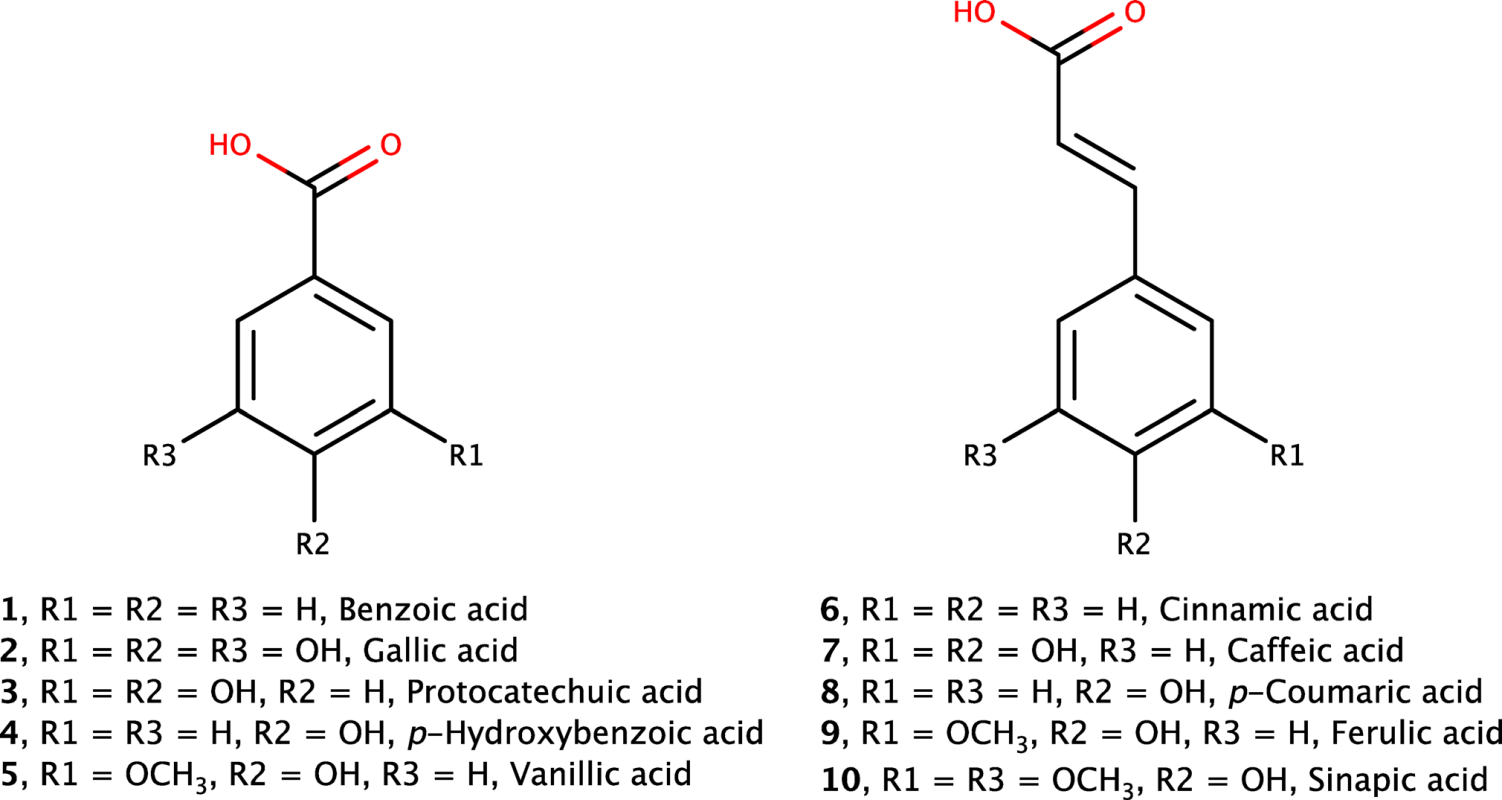
Structures of benzoic acid (1), common hydroxybenzoic acids (2–5) and common hydroxycinnamic acids (6–10)

Phenolic acids are a class of secondary metabolites, part of a large group of phenolic compounds, widely distributed in the plant kingdom. They are considered important constituents of food, contributing to taste, colour and nutritional properties. Based on their chemical structure, phenolic acids can be classified into benzoic acid and cinnamic acid derivatives (Fig. 1). Hydroxycinnamic acids (HCAs) are synthetized in plants from phenylalanine via cinnamic acid or directly from tyrosine by tyrosine ammonia-lyase, producing the simplest hydroxycinnamic acid, *p*-coumaric acid, which can be further synthetized into caffeic, ferulic and sinapic acids [1, 2].

### Sources and intake of hydroxybenzoic acids (HBAs)

The main hydroxybenzoic acids (HBAs) are gallic acid (3,4,5-trihydroxybenzoic acid), protocatechuic acid (3,4-dihydroxybenzoic acid), and *p*-hydroxybenzoic acid (4-hydroxybenzoic acid) [3]. Particularly high contents of *p*-hydroxybenzoic acid-*O*-glucoside have been found in spices and herbs belonging to the Apiaceae family: anise 730–1080 mg/kg fresh weight (FW), caraway up to 42 mg/kg FW, fennel up to 106 mg/kg FW, and coriander up to 30 mg/kg FW [4]. In addition, star anise (in the Schisandraceae family) contains *p*-hydroxybenzoic acid-*O*-glucoside 730–840 mg/kg FW. The concentration of hydroxybenzoic acids in fruits and vegetables is generally low and they are mostly present in conjugated forms [3, 5]. For example, gooseberries, blackberries, and raspberries contain *p*-hydroxybenzoic acid-*O*-glucoside 9–14 mg/kg FW, 4–18 mg/kg FW, and 32–56 mg/kg FW, respectively, whereas protocatechuic acid-4-*O*-glucoside contents varies from trace amount to 6 mg/kg FW in the same berries, and gallic acid-4-*O*-glucoside up to 3 mg/kg FW. Blueberries have a different HBA profile, as they contain mainly *p*-coumaroylquinic acid (1860–2080 mg/kg FW) and only 4–5 mg/kg, 3–6 mg/kg, and 2–9 mg/kg FW of *p*-hydroxybenzoic acid-*O*-glucoside, protocatechuic acid-4-*O*-glucoside, and gallic acid-4-*O*-glucoside, respectively [4]. Marionberries and boysenberries have been reported to contain gallic acid 30 mg/kg and 90 mg/kg FW, respectively [6]. In addition, chestnuts contain high amounts of gallic acid (3460–9070 mg/kg FW) [7]. Onion peel contains HBAs, mainly protocatechuic acid-4-*O*-glucoside in a concentration of 91 mg/kg FW [4]. Hydroxybenzoic acids can also be complexed to large structures: gallic acid can form oligomers known as gallotannins, while ellagic acid, the dilactone of hexahydroxydiphenic acid, is a constituent of hydrolysable ellagitannins, abundant in, e.g., *Rubus* berries (2630–3300 mg/kg FW), pomegranate juice (1500–1900 mg/l), and walnuts (16,040 mg/kg FW) [8].

### Sources and intake of hydroxycinnamic acids (HCAs)

HCAs are widely distributed in plants [2], occurring in nature generally as esters (formed by conjugation with quinic, shikimic, and tartaric acids, flavonoids, or carbohydrates), or as amides (formed by conjugation with amino acids or amines) [2, 9, 10]. High concentrations of HCAs can be found in many food products, such as coffee, tea, wine, cocoa, fruits, vegetables, and cereals. Their exact level varies among plant food varieties [11]. Free and simple HCA esters are abundant in fruits and vegetables, while bound HCA derivatives are more common in grains. HCA-derived amides are mostly found in coffee and cocoa [2]. Caffeic acid and coumaric acid are abundant in fruits, such as apples, pears and berries, representing between 75 and 100% of the total HCA content of most fruits [9]. Some of the richest sources of caffeic acid include wild blueberry (1470 mg/kg FW), coffee (870 mg/kg FW), carrot (260 mg/kg FW), plum (234 mg/kg FW), and aubergine (210 mg/kg FW) [2, 12]. Caftaric acid (2-caffeoyltartaric acid), a caffeic acid derivative, is an important phenolic compound in wine (6–73 mg/l in white wine, 46–141 mg/l in red wine) [2, 13, 14]. Among the most abundant free HCA derivatives are chlorogenic acids, formed by the conjugation of quinic acid with certain HCAs, most commonly caffeic, *p*-coumaric, or ferulic acid [10, 15, 16]. The main subgroups of chlorogenic acids are caffeoylquinic, dicaffeoylquinic, feruloylquinic and *p*-coumaroylquinic acids [10]. Chlorogenic acids are found in many types of fruits and in high concentrations in coffee (depending on climatic and processing conditions, degree of roasting, grinding and barista procedures) [2, 9, 17, 18]. Green coffee beans contain the largest amounts of chlorogenic acids, and some of these are transformed into their corresponding chlorogenic acid lactones during the roasting process; the chlorogenic acid content of roasted coffee beans varies according to the degree of roasting in the range of 2350–80,000 mg/kg DW [10] and in espresso coffee 890–8130 mg/l [18]. Chlorogenic acids are also found in vegetables, yerba mate, and tea [2, 19]. Ferulic acid is by far the most abundant and common HCA in cereal grains, which are the main dietary source of the compound (intake ranging from 91.5 to 320 mg from wheat bran, rye bran, or whole-grain rye bread in the reported interventions (Table 1; [20–22]), and in whole maize grain [23]. Flavonoid conjugates of ferulic acid and other HCAs have been found in *Brassica* vegetables (calculated range 200–3600 mg/kg DW) [24–26]. Ferulic acid in its free form exists in tomatoes and beer [9]. Several ferulic acid derivatives, esterified to cell wall polymers in cereal bran, as well as ferulic acid dimers, trimers and tetramers, have been described [27]. The most important sources of sinapic acid are *Brassica* vegetables, and several conjugated forms of sinapic acid with flavonoids have been described in these vegetables [24–26]. Whole grain cereals are also important sources of sinapic acid [2], which is shown as higher intake of sinapic acid from whole-grain cereals (17–37.3 mg; Table 1) than from white wheat bread (2.2 mg; Table 1) [21, 22]. Coumaric acid (especially *p*-coumaric acid) is also an abundant HCA in most fruits and cereals. It is abundant in strawberries (1110 mg/kg DW), other berries (14–950 mg/kg DW), peanuts (1030 mg/kg FW), rye bran (140 mg/kg FW), and red wine (50 mg/kg FW) [2].

**Table 1:** Mean concentrations and standard deviations of phenolic acid metabolites found in plasma and urine, after the consumption of foods rich in phenolic acids

Food source	Compounds (mg)	Total consumed (mg)	Metabolites	Max conc. in plasma (nM)	Total in 24-h urine (mg)	Number of subject and gender	References
White wheat bread fortified with bioprocessed rye bran	Ferulic acid Sinapic acid *p*-Coumaric acid	160.6 37.3 5.6	Ferulic acid Sinapic acid *p*-Coumaric acid	–	1.66 ± 0.51 0.23 ± 0.09 0.02 ± 0.01	6 males 9 females	[21]
White wheat bread fortified with native rye bran	Ferulic acid Sinapic acid *p*-Coumaric acid	122.7 30.2 5.2	Ferulic acid Sinapic acid *p*-Coumaric acid	–	0.45 ± 0.15 0.12 ± 0.06 0.02 ± 0.01	6 males 9 females	[21]
White wheat bread	Ferulic acid Sinapic acid *p*-Coumaric acid	8.4 2.2 0.3	Ferulic acid Sinapic acid *p*-Coumaric acid	–	0.27 ± 0.10 0.06 ± 0.03 0.01 ± 0.01	6 males 9 females	[21]
Rye bread	Ferulic acid Sinapic acid *p*-Coumaric acid	91.5 21.1 3.6	Ferulic acid Sinapic acid *p*-Coumaric acid	–	0.33 ± 0.14 0.07 ± 0.06 0.01 ± 0.01	6 males 9 females	[21]
Whole wheat bread containing native bran	Ferulic acid Sinapic acid *p*-Coumaric acid Vanillic acid	320 17 5.4 4.9	Ferulic acid Sinapic acid *p*-Coumaric acid Vanillic acid	880 (150)* n/d n/d 110 (30)*	9.9 (1.9)* 1.1 (0.8)* 0.1 (0.07)* 5.0 (1.3)*	8 males	[22]
Whole wheat bread containing bioprocessed bran	Ferulic acid Sinapic acid *p*-Coumaric acid Vanillic acid	220 17 5.4 4.9	Ferulic acid Sinapic acid *p*-Coumaric acid Vanillic acid	2700 (630)* n/d n/d 250 (180)*	21.4 (8.9)* 2.7 (0.7)* 0.09 (0.05)* 8.2 (5.7)*	8 males	[22]
Rye bran bread	Ferulic acid	10.2	Ferulic acid	–	4.82 ± 3.46	18 females	[55]
Wholegrain wheat bread	Ferulic acid	87	Ferulic acid-4′-*O*-sulfate Dihydroferulic acid-4′-*O*-sulfate Dihydroferulic acid-*O*-glucuronide	84.3 ± 16.6 9.2 ± 1.4 n/d	P P P	8 males 7 females	[87]
Commercial wheat bread enriched in aleurone fraction	Ferulic acid	43	Ferulic acid-4′-*O*-sulfate Dihydroferulic acid-4′-*O*-sulfate Dihydroferulic acid-*O*-glucuronide	55.5 ± 9.4 9.5 ± 3.5 n/d	P P P	8 males 7 females	[87]
Commercial wheat bread enriched in aleurone fraction	Ferulic acid	87	Ferulic acid-4′-*O*-sulfate Dihydroferulic acid-4′-*O*-sulfate Dihydroferulic acid-*O*-glucuronide	76.6 ± 9.1 11.9 ± 1.9 n/d	P P P	8 males 7 females	[87]
Commercial breakfast cereal (85% wheat bran)	Vanillin *t*-*p*-Coumaric acid *trans*-Sinapic acid *t*-Ferulic acid *cis*-Ferulic acid 8-5-diFA (open form) 5-5-diFA 8-*O*-4-diFA 8-5-Benzofuran-diFA	2.72 ± 0.40 5.44 ± 0.45 19.60 ± 1.73 259.10 ± 15.63 9.22 ± 4.96 4.94 ± 0.18 6.43 ± 0.24 14.46 ± 1.92 2.85 ± 0.75	Ferulic acid Sinapic acid	150 to 210 ~ 10 to 40	8.10 ± 3.34 0.54 ± 0.13	3 males 3 females	[29]
Cranberry juice	Cinnamic acid *p*-Coumaric acid Caffeic acid Ferulic acid Chlorogenic acid Benzoic acid 2-Hydroxybenzoic acids 3,4-Dihydroxybenzoic acid Gallic acid Vanillic acid	1.0 6.9 1.2 0.0 5.2 7.8 0.1 1.1 0.1 1.0	*Selected metabolites* Cinnamic acid *p*-Coumaric acid Caffeic acid Ferulic acid Chlorogenic acid Ferulic acid-4′-*O*-sulfate Ferulic acid-4′-*O*-glucuronide Dihydroferulic acid Benzoic acid 2,3-Dihydroxybenzoic acids Protocatechuic acid Syringic acid Vanillic acid Vanillic acid-4-*O*-sulfate Isovanillic acid 4-Methylgallic acid-3-*O*-sulfate Hippuric acid α-Hydroxyhippuric acid 2-(4-Hydroxyphenoxy)propionic acid Homovanillic acid 3,4-Dihydroxyphenyl acetic acid 3-Hydroxyphenyl acetic acid 4-Hydroxyphenyl acetic acid	123 ± 34 6 ± 1 1 ± 1 57 ± 12 5 ± 2 2268 ± 794 165 ± 29 304 ± 122 2169 ± 608 12,024 ± 4055 109 ± 45 8 ± 6 410 ± 115 1054 ± 274 220 ± 44 275 ± 82 42,926 ± 12 282 2943 ± 587 n.d. 511 ± 165 476 ± 138 615 ± 360 1849 ± 724	*nmol* n/d 36 ± 9 75 ± 22 n/d 52 ± 25 1055 ± 259 109 ± 20 524 ± 368 4141 ± 427 8432 ± 2176 944 ± 162 249 ± 31 423 ± 136 288 ± 87 1238 ± 229 297 ± 74 69,717 ± 13,686 74,538 ± 20,636 453 ± 152 6640 ± 1472 1597 ± 297 4384 ± 922 10,152 ± 3161	10 males	[63]
Coffee	Caffeoylquinic acid Feruloylquinic acid Dicaffeoylquinic acid	256 37 42	Dihydroferulic acid Dihydrocaffeic acid Caffeic acid Ferulic acid Isoferulic acid	P (major) P (major) P (minor) P (minor) P (minor)	–	4 males 5 females	[62]
Instant coffee	Caffeoylquinic acids Feruloylquinic acids Caffeoylquinic acid lactones Dicaffeoylquinic acids *p*-Coumaroylquinic acids	95.31 ± 1.74 24.68 ± 1.95 19.17 ± 1.65 6.51 ± 0.62 2.30 ± 0.03	3-*O*-Caffeoylquinic acid lactone-O-sulfate 3-*O*-Feruloylquinic acid Caffeic acid-3-*O*-sulfate Ferulic acid-4-*O*-sulfate Dihydroferulic acid Dihydroferulic acid-4-*O*-sulfate Dihydrocaffeic acid-3-*O*-sulfate… And others	27 ± 3 16 ± 2 92 ± 11 76 ± 9 385 ± 86 145 ± 53 325 ± 99	1.1 ± 0.1 1.2 ± 0.1 6.4 ± 0.8 11.1 ± 1.6 9.7 ± 2.0 12.4 ± 3.4 37.1 ± 8.2	8 males 3 females	[61]
Yerba mate infusion	Caffeoylglucose isomers Caffeoylquinic acids Coumaroylquinic acids Feruloylquinic acids Caffeoylquinic lactones Dicaffeoylquinic acids Caffeoylferuloylquinic acids Flavonols	3.5 ± 0.2 44.5 ± 0.8 0.5 ± 0.1 3.2 ± 0.1 2.7 ± 0.2 22.4 ± 0.5 0.5 ± 0.1 3.3 ± 0.3	Coumaroylquinic acid Caffeic acid 3-sulfate Ferulic acid 4-glucuronide Ferulic acid 4-sulfate Others Microbial metabolites	Traces 37 ± 36 11 ± 4 19 ± 9 n/d	0.179 ± 0.033 1.261 ± 0.330 1.098 ± 0.278 2.234 ± 0.701	7 males 5 females	[19]
Polyphenol rich diet Response after 8 week intervention *N* = 34	Polyphenols Flavones Flavonols Flavanols Flavanones Anthocyanidins Isoflavones Phenolic acids	mg 2868 7.6 223 1194 102 111 0.02 1245	*Phenolic acid microbial metabolites in 24-h-urine* 3-(3′,4′-Dihydroxyphenyl)propionic acid 3-(3′-Hydroxyphenyl)propionic acid 3-(4′-Hydroxyphenyl)propionic acid 2-(3′,4′-Dihydroxyphenyl)acetic acid 2-(3′-Hydroxyphenyl)acetic acid 3,4-Dihydroxybenzoic acid 3,5-Dihydroxybenzoic acid 3,4-Dimethoxybenzoic acid 3-Hydroxybenzoic acid 4-Hydroxybenzoic acid Benzoic acid 4-Methylcatechol Gallic acid Ferulic acid Sinapic acid Caffeic acid 4-Coumaric acid Vanillic acid Hippuric acid	–	*nmol/mg creatinine* 0.14 ± 0.16 0.12 ± 0.12 0.002 ± 0.000 0.10 ± 0.07 0.33 ± 0.25 0.03 ± 0.01 0.02 ± 0.01 0.01 ± 0.01 0.01 ± 0.01 0.06 ± 0.04 0.06 ± 0.13 0.07 ± 0.07 0.004 ± 0.000 0.13 ± 0.08 0.10 ± 0.09 0.06 ± 0.04 0.003 ± 0.000 0.11 ± 0.07 23 ± 20	16 males 19 females	[85]

*Conc* concentration, *n/d* not detected, *P* present, *asterisk* values are medians, *IQR* interquartile range (middle 50%), *double asterisks* largest standard deviation from the individual components in the sum

### Bioavailability of phenolic acids

Phenolic acids in cereals are present both in free form and bound by ester bonds to arabinoxylan chains or by ether bonds to lignin. Most of the dietary fibre-bound phenolic acids in cereals are esterified to the cell walls. Ferulic acid can dimerize into dehydrodimers that cross-link arabinoxylan chains and/or lignins and thereby affect the physical properties of cell walls and consequently the solubility and degradability of arabinoxylans in the colon [28]. The main ferulic acid dehydrodimers identified in barley and oats are 8-*O*-4-diferulic acid, 5,5′-diferulic acid and 8,5-diferulic acid (benzofuran form). The intake of dimers was observed by Kern et al. [29], but only a minor part of ferulic and sinapic acids were detected from the urinary excretion (Table 1). The content of dehydrodimers and ferulic acid seems to correlate with the content of insoluble dietary fibre and was found to be 3599 ± 69 and 3658 ± 170 mg/kg in oats and barley, respectively, with much lower contents detected in soluble dietary fibre fractions of the two cereals (38 ± 5 and 69 ± 10 mg/kg), respectively [28]. The dehydrodimers are abundant also in the insoluble dietary fibre of maize (12 596 ± 184 mg/kg^− 1^), wheat (2375 ± 36 mg/kg), spelt (2601 ± 59 mg/kg), rice (4042 ± 59 mg/kg), wild rice (2840 ± 130 mg/kg), rye (3647 ± 132 mg/kg) and millet (5693 ± 231 mg/kg), while the content in the soluble dietary fibre is only 59 ± 3 mg/kg in maize, 184 ± 14 mg/kg in wheat, 233 ± 43 mg/kg in spelt, 83 ± 3 mg/kg in rye, and 46 ± 9 mg/kg in millet, lacking completely in rice and wild rice [28]. Bunzel et al. [28] showed also different profiles in the diferulate species in these crops, indicating varying percentages of 8-5′ isomers in total dehydrodiferulates 37% (in maize soluble fraction) to 54% (in rye insoluble fraction), 8-8′-dehydrodiferulates from 16% in barley insoluble dietary fibre to 46% in spelt soluble fraction, 5-5′-dehydrodiferulates varying from 7% in spelt soluble dietary fibre to 25% in maize insoluble dietary fibre fraction [28]. Although ferulic acid dehydrodimers are common, dehydrodisinapic acids and sinapate–ferulate crossed dehydrodimers are less common but have also been identified in cereal dietary fibre [30].

The biological activities of phenolic acids depend on their bioavailability in vivo. Most phenolic acids in cereals (> 99%) are present in their bound form, and thus they are poorly bioavailable [31]. In berries, the content of bound phenolic acids varies between > 90% in raspberry and cowberry, 70% in strawberry, 60–70% in bilberry, lingonberry, and cloudberry, and 10–30% in rowanberry and blueberry. Among fruits, the highest contents of phenolic acids are found in dark plum, cherry, citrus fruits, red grape, and some apple varieties. However, the contents in these fruits (< 300 mg/kg FW) were clearly lower than in the berries with the highest phenolic acid content (590–1030 mg/kg FW). The contents of phenolic acids in beverages varies widely, ranging from 0 mg/kg in pear cider, 160 mg/kg FW in apple juice, 300–360 mg/kg FW in green and black tea to 970 mg/kg FW in coffee and 1520 mg/l in yerba mate [19, 32]. Most of the phenolic acids in beverages are liberated after hydrolysis, indicating that nearly all phenolic acids are conjugated or bound in the original ingredients.

### Impact of food matrix and processing on the bioavailability of phenolic acids

The bioavailability and thus the phenolic acid concentrations in the body fluids measured in intervention trials are affected by the food matrix and its processing. A good example is the processing of cereal matrix, which contains higher concentrations of bound phenolic acids (1300–1400 mg/kg FW in whole wheat and rye flour, > 4000 mg/kg FW in wheat and rye bran) compared to their free forms (< 20 mg/kg FW) [11]. Many preliminary processing techniques remove phenolic acids or increase the amount of free phenolic acids in cereal food products. Dehulling reduced the total content of phenolic acids considerably in oat groats, pearled barley, and rye flour [33, 34], whereas milling and air-classification increased the content of bound phenolic acids from the levels present in the whole flour [35, 36]. Phenolic acids embedded in the bran matrix can be released during bioprocessing using enzymes alone, such as ferulic acid esterase, xylanases or cellulases [37–40] or by combining enzymes with sourdough fermentation [31, 41–43]. Germination is a traditional means of bioprocessing alone or in combination with dough fermentation with yeast, which enhance the release of free phenolic acids in dough [44, 45].

Bread-making includes several fundamental operations, namely mixing, fermentation and baking, which are indispensable for producing an attractive bread. Fermentation of the dough contributes to an increase in phenolic acids and the mechanism proposed is via the structural breakdown of the cell wall matrix by degrading enzymes present in both grains and microbes such as xylanases and esterases [46]. Dough mixing causes an overall decrease in bound ferulic acid, sinapic acid, and caffeic acid in various grains, increasing the amount of free ferulic acid significantly up to fivefold from the initial level [20, 47, 48]. Yu and Beta [49] found an increase (103–109%) in the contents of free ferulic acid and *p*-hydroxybenzoic acids in bread crust after baking in the oven, suggesting that some free phenolic acids are thermally labile. However, in the crumb, the levels of bound phenolic acids increased instead [49]. Other thermal processes include extrusion and infrared heating, which may have a varying effect on the content of free phenolic acids. For example, extrusion with high temperature and pressure increased the content of free phenolic acids in pearled barley by 72%, whereas milder conditions by infrared heating and flaking of the same material did not have any effect [33]. In vitro models have been used to study the impact of bioprocessing on the release of phytochemicals including phenolic acids [50–52].

There are only few in vivo studies and on the degree of processing, including animal studies [53, 54] and human interventions [21, 22, 55], all of which showed a variation in the urinary excretion of HCAs or their metabolites. Bioprocessing showed a significant role on the bioavailability of ferulic acid only, while *p*-coumaric acid contents did not change neither in whole grain rye nor in whole-grain wheat [21, 22, 56, 57]. Table 1 gives examples of selected food items, their phenolic acid consumption levels, and bioavailability as HCAs after deconjugation before analysis (Table 1; [21, 22, 55]).

In conclusion, to assess the dietary intake of phenolic acids and their conjugates, accurate and wide-scale analysis is crucial to determine the diversity and the contents of compounds in food stuffs. For non-traditional wheat species, such as einkorn, emmer, spelt, and pigmented cultivars, a significant gap remains concerning the release properties of their matrix; these crops might deserve a more substantial role in human nutrition, potentially affecting health responses.

## Metabolism of phenolic acids

When phenolic acids are consumed, they undergo substantial metabolism by tissues, organs and colonic microbiota. In this section, the metabolic routes and interplay between the host and residing microbiota are discussed.

Free phenolic acids can be released from food and beverage matrices in the stomach, the muscles of which reduce food particle size and further enhance the release of phenolic compounds and their absorption [58–60]. Table 1 shows the impact of food matrix in the bioavailability: the absorption of phenolic acids from beverages (cranberry juice, coffee, and yerba mate [61–63]) occurs at a higher extent than from solid food matrices (whole grain cereals) (Table 1). Since a high proportion of HCAs is conjugated or bound to dietary fibre matrix, the majority of these compounds reach the colon, and their bioavailability requires the activity of degradation enzymes in intestinal tissues and microbiota [64–68]. Hydrolysis by intestinal or microbial esterases is probably the major route for release of free HCAs from their conjugation in vivo. After deconjugation, the released phenolic acids can be absorbed across the gastrointestinal barrier and enter the peripheral blood circulation [65].

### Metabolism by the host

Absorbed dietary phenolic acids can be perceived by the body as xenobiotic substances and thus they undergo metabolism to facilitate their removal. Xenobiotic metabolism is a multi-organ process, starting in the upper intestinal epithelia and largely continuing in the lower intestine and liver, as well as in peripheral tissues, such as kidneys and adipose tissue. Hepatic enzymes transform molecules by adding or removing hydroxyl groups (phase I) and conjugating them to other molecules (phase II) to increase their water solubility, thus enhancing their excretion in urine. HCAs undergo glucuronidation and/or sulfation, or are oxidized into benzoic acid derivatives, which are finally glycinated into hippuric acid derivatives [1, 69–71]. Table 1 shows maximal concentrations in plasma (nM) and total 24-h urinary excretion as mg or as nmol of hepatic metabolites of HCAs, which in some studies are deconjugated before analysis [21, 22, 29, 55] and expressed as free phenolic acids, and in others as sulfate and glucuronide conjugates of ferulic acid.

Xenobiotic metabolism is also subjected to individual responses. The enzymes of detoxification, responsible for phase I and II metabolism and phase III transport [72], may be expressed differently upon nutritional stimuli or genetic polymorphisms, causing interindividual variation [73]. One of the major enzyme systems that handles the capacity of an organism to metabolize xenobiotics, are the cytochrome P450 monooxygenases [74]. The expression of CYP2D6 (debrisoquine hydroxylase) and CYP2C19 (mephenytoin hydroxylase) genes may vary up to 1000-fold between individuals and thus lead to individuals considered as poor or extensive metabolizers [73]. Genetic polymorphisms have been described for most drug metabolizing enzymes, such as genes involved in acetylation and oxidation. Slow acetylator phenotypes occur in habitants of Europe and North America (40–70%), while in Asia Pacific, only 10–30% of the residents are slow acetylators. The cytosolic *N*-acetyltransferase in the liver, responsible for the transfer of acetate from acetyl-CoA to the substrate, is encoded by the *NAT1* and *NAT2* genes. The slow acetylator phenotype is related to a reduction of 10–20% of NAT2 protein in the liver, due to the existence of *NAT2* alleles with decreased functionality [73].

### Metabolism by gut microbiota

The role of gut microbiota in the biotransformation of phytochemicals, phenolic acids among them, is widely acknowledged, causing the formation of food-derived metabolites in the circulation and excreted in the urine. Table 1 shows some examples of the analysed microbial metabolite profiles from human interventions. However, in vivo results show large interindividual variation, and when the metabolite profile contains many different metabolites with high interindividual variation, any differences between the study groups become more difficult to detect.

Since the biotransformation capacity of the gut microbiota is extensive, in vitro studies have attempted to identify colonic metabolites of phenolic acids. Braune et al. [75] studied the in vitro anaerobic degradation of isolated ferulic acid dimers, 8-*O*-4-diferulic acid (C–*O*–C-bond) and 5-5-diferulic acid (C–C-bond), using human faecal suspension as an inoculum. They showed that the biotransformation of C–*O*–C-dimers started with the release of ferulic acid (which was converted to hydroxylated phenylpropionic acids) and 3-(4-hydroxy-3-methoxyphenyl)pyruvic acid (which was a precursor to hydroxylated phenylacetic and phenyllactic acids) [75]. The further formation of 3-(3-hydroxyphenyl)propionic acid and its subsequent dehydroxylation to 3-phenylpropionic acid has been shown for ferulic acid and caffeic acid derivatives [57, 76]. In addition, C–C-bonded 5-5-diferulate had conversions in the attached units without degradation of the C–C-bond. Among these conversions were series of demethylations and double bond reductions, which resulted in different combinations of two moieties of caffeic acid, dihydrocaffeic acid (3-(3ʹ,4ʹ-dihydroxyphenyl)propionic acid), ferulic acid or dihydroferulic acid (3-(3ʹ-methoxy-4ʹ-hydroxyphenyl)propionic acid) attached with the C–C bond [75].

The formation of vanillic acid has been suggested to occur in the colon from the main microbial metabolite of ferulic acid, dihydroferulic acid, by β-oxidation [50, 63]. Ferulic acid can also be degraded to caffeic acid and by reduction to *p*-coumaric acid and cinnamic acid. The β- or α-oxidation of the aliphatic side chain leads to the accumulation of benzoic acid derivatives that is perhaps the most common intermediate of anaerobic degradation [77–79]. Figure 3 shows also examples of the microbial metabolites, phenylpropionic or phenylacetic acids with different patterns of substitution. It is worthy of a note that benzoic acid derivatives, including hydroxybenzoic acids and their glycinated hepatic metabolites, hippuric acids, are a substantial part in the circulating and excreted metabolite profile, not only of phenolics acids, but also main dietary flavonoids [12].

It is difficult to distinguish the actions of liver from those of the colon without studying the metabolism using in vitro colon and hepatic models, since in vivo studies show their concerted action and the impact of enterohepatic circulation. Human primary hepatocytes are capable of converting phenylpropionic acid to ferulic acid, indicating the presence of post-colonic hepatic metabolites and returning the structural transformation back to original precursor structures [80]. Enterohepatic circulation has shown to contribute to the low diurnal variation of the colonic metabolite concentrations in the blood [81, 82], and also contributing to their long residence time. Pharmacokinetic studies have shown that microbial phenolic acid metabolites have a 24–48 h residence time in the bloodstream after a single dose of their parent compounds [83, 84]. Phenolic acids are excreted in urine mainly in their free form [83], but it is possible that liver has a role in formation of the post-colonic metabolites [80]. Phenolic acid and phenolic acid microbial metabolite levels in urine are on low or high micromolar level [85], whereas in plasma they range from low to high-nanomolar concentrations [12, 84, 86]. In peripheral tissues, the concentrations can be anticipated to be even lower.

Figure 3 shows benzoic acid derivatives and HCAs after reduction of the double bond in the side chain and conversion to dihydroferulic acid derivative [63, 87] and also dihydrocaffeic acid [61, 62], which can be expressed also as phenylpropionic acid derivatives, indicating microbial metabolism. It is noteworthy that HCA metabolites are shared with those of flavonoids and can together have a bigger impact on in human body as a pool of metabolites [80, 85]. As the metabolites described above and their hepatic conjugates are found in plasma and urine, they circulate through the body and may exhibit both local and systemic effects.

In conclusion, interindividual variation is affected by several sites of metabolism, tissues and the intestinal microbiota, causing a diverse metabolite profile. The overall metabolite pool, which circulates in the body, mediates the health benefits of a diet, which may be difficult to attribute to a single food item, study group, or compound class due to high individual variation and interlinked metabolic pathways. The ecology of gut microbiota and variation of the functional genes of the microbes may have a role in interindividual variability in the circulating metabolites and in their health benefits.

## Health benefits related to intake of phenolic acids

The potential preventive effect of HCAs on several chronic diseases has been widely reviewed in the recent years. HCA derivatives, in particular chlorogenic acids, have been shown to exert health benefits in the management of obesity, cardiovascular diseases, type 2 diabetes mellitus, and metabolic syndrome [2, 88–92], while some antioxidant and neuroprotective effects have also been reported [93–95]. Nevertheless, current evidence is still inconsistent and insufficient to support robust health claims, mostly because the co-occurrence of other plant bioactives (e.g., caffeine and hydroxyhydroquinone in coffee, fibre in wholegrain products, and flavonoids in berries and fruits) in the main dietary sources of HCAs hinders the elucidation of the putative health benefits exerted by these compounds. Moreover, the interindividual variability in the physiological response to consumption of HCAs is almost unknown for most of the biomarkers of cardiometabolic health [96].

HCAs have shown a series of biological activities related to the prevention of cardiovascular diseases, diabetes, and metabolic syndrome in cell and animal studies [2, 89–92, 97–99], but evidence in humans is rather limited. HCAs may enhance cardiovascular health by exerting blood pressure-lowering effects and by acutely improving the endothelial function [89, 100, 101]. Some studies suggest that chlorogenic acids may cause significant reductions in systolic and diastolic blood pressure [100, 102, 103], but other studies have instead reported a lack of significant blood pressure reduction [104–106]. The differences could be attributed to the antihypertensive effects of chlorogenic acids only occurring in subjects with mild hypertension and not in normotensives [88, 107]. Moreover, the decrease in blood pressure upon consumption of caffeoylquinic acids from coffee could be inhibited by the presence of hydroxyhydroquinone in brewed coffee. Hydroxyhydroquinone is generated during the roasting of coffee beans, and it is a compound to which prohypertensive effects have been attributed [104]. In addition, dose–response effects of HCAs on blood pressure are not clear [88]. These controversies and lack of robust evidence make the development of health claims or guidelines on the effects of HCA-rich diet on reduced blood pressure extremely difficult.

The acute effects of HCAs on endothelial function are also contradictory. While some human studies indicated a lack of effect of 400 mg of chlorogenic acid (5-caffeoylquinic acid) or coffee containing different amounts of HCAs on flow-mediated dilation (FMD) [106, 108], a recent intervention trial by Ward et al. [105] in healthy men and women reported significant effects of chlorogenic acid, at doses of 450 and 900 mg, on mean post-ischaemic FMD response. To further complicate the picture, in a similar study conducted only in healthy men, Mills et al. [101] observed improvements in vascular function after pure chlorogenic acid intake, but only at 450 mg and not at 900 mg intake level. Interestingly, these authors also investigated the impact of coffee intake, matching for caffeine, but differing in the content in chlorogenic acids (89 and 310 mg), on FMD response. A bi-phasic FMD response was observed regardless of the intake level, closely in concomitance with the appearance of HCA metabolites in plasma [101]. One of these metabolites peaking in plasma in parallel to the vascular function improvement was ferulic acid 4ʹ-*O*-sulfate [101], which has recently demonstrated to elicit a concentration-dependent vasorelaxing effect ex vivo [109]. Therefore, further studies are needed to establish a dose–response relationship and to determine the molecular species and their adequate plasma levels to demonstrate the FMD response.

Regarding body weight, Thom observed that the consumption of chlorogenic acid-enriched instant coffee in overweight subjects for 12 weeks significantly reduced body mass and body fat when compared with the use of normal instant coffee [110]. Slight changes in weight were also observed in mildly hypertensive adults when testing coffees deprived of hydroxyhydroquinone and containing different amounts of chlorogenic acids for 4 weeks [107]. On the contrary, chlorogenic acid-rich coffee consumed daily for 4 weeks did not lead to a significant body weight reduction in pre-obese [111] and healthy subjects [112]. An effect of chlorogenic acid consumption on body mass index (BMI) was observed by Watanabe et al. [102] in patients with mild hypertension.

Other biomarkers related to cardiometabolic health have been less investigated. A study reported that the lipid profile of healthy adults did not change after a daily consumption of 400 ml of coffee containing a medium (420 mg) or high (780 mg) content of HCAs for 8 weeks [106]. Another trial, which controlled the caffeine intake, indicated that the consumption of coffees containing different amounts of chlorogenic acids attenuated the effects of short-term fructose-induced liver insulin resistance in healthy men [113]. Ochiai et al. [104] showed that coffee chlorogenic acids might decrease urinary isoprostane levels, suggesting a reduced oxidative stress in mild hypertensive Japanese adults with vascular failure and not taking any antihypertensive drugs. The same effect, lowered isoprostane levels, was found in subjects suffering from metabolic syndrome symptoms and using polyphenol-rich diet for 8 weeks, showing increased urinary levels of microbial phenolic acid metabolites, which are shared by the HCAs [85, 114]. Therefore, it may be difficult to draw conclusions on the role of a single compound group, such as HCAs in systemic disorders such as lipid metabolism, glucose metabolism, insulin resistance, and inflammation based on the limited insights achieved so far in controlled human studies in chronic conditions. However, currently, a systematic review and meta-analysis of randomized controlled trials is being carried out to shed light on the interindividual variability in response to the impact of HCAs on cardiometabolic biomarkers [115]. This work will possibly help to better understand the protective features of HCAs in different population subgroups, but further ad hoc designed clinical trials are required, in our opinion, to tackle the health benefits of HCAs.

Acute studies dealing with postprandial responses seem to indicate that chlorogenic acid intake may attenuate early glucose absorption and insulin response [116, 117] and that this effect does not seem to be mediated by incretin hormones (glucagon-like peptide-1, GLP-1, and glucose-dependent insulinotropic polypeptide, GIP) in overweight men [118]. In another study, daily consumption of 329 mg of coffee chlorogenic acids increased postprandial fat utilization in healthy humans [112], mainly when hydroxyhydroquinone was not present in the brew [119].

The chemopreventive properties of HCAs have been evaluated mainly in animal and cell models [91]. In humans, the main insights in the chemopreventive activity of HCAs come from observational studies indicating, for instance, an inverse association of wholegrain phenolics with the incidence of colorectal cancer [120], however, results from these studies are affected by several confounding factors, the first being the practical impossibility to fully adjust for fibre intake. Moreover, it has been suggested that chlorogenic acids in coffee may play a role in protecting DNA integrity [121] and in the induction of chemopreventive phase II enzymes via the Nrf2/antioxidant-response element (ARE) detoxifying pathway [122]. This latter effect could vary significantly among individuals, due to the existence of genetic polymorphisms in the *Nrf2* gene [122].

The biological properties of HCAs in the framework of brain function have been scarcely investigated [93, 95]. To date, only a few studies have been conducted in humans to address the cognitive and mood effects associated to their intake. In an acute study in healthy elderly subjects, Cropley et al. [123] reported that coffee chlorogenic acids may modulate brain function by improving some mood and behavioural processes. In another study, using a decaffeinated green coffee blend and pure 5-caffeoylquinic acid, Camfield et al. [124] assessed several cognitive and mood outcomes and concluded that the improvements observed in mood, but not in cognitive function, could be partially attributable to coffee chlorogenic acids. Further work is required to tackle the effects of HCAs consumption on human brain function.

## Interindividual variability in health responses

The interindividual variability in health responses to phenolic acid-rich foods has been scarcely studied, although some insights have been gained. A polyphenol-rich diet was shown to significantly reduce the markers of dyslipidaemia (triglycerides and VLDL) and oxidative stress (8-isoprostane urinary excretion) in 86 overweight or obese individuals despite of a large variability in the responses [114]. However, the variability was not associated with a specific factor, nor was the diet assessed for the intake of particular classes of polyphenols, including phenolic acids and flavonoids. Artichoke leaf extract, containing mainly HCAs (chlorogenic acid, cynarin, and caffeic acid), sesquiterpene lactones, and flavonoids (e.g., luteolin), decreased LDL-C levels only in men with a certain genotype of Taq IB in the *CETP* gene (cholesteryl ester transfer protein) [125]. The effect is likely attributable to the (poly)phenols present in artichoke, but the contribution of phenolic acids remains unclear, since both chlorogenic acid and luteolin can inhibit LDL oxidation in vitro [126]. In another RCT study by Gavrieli et al., coffee delayed the postprandial glucose response more in females and overweight or obese participants compared to men and participants with normal weight, respectively [127]. A randomized crossover study, where coffee containing green and roasted beans was consumed by 52 volunteers, found decreased levels of lipid markers (LDL-C, VLDL-C, and triglycerides) only in the hypercholesterolemic group (*n *= 27), but not in the normocholesterolemic one (*n *= 25) [128]. The same research group also found a decrease in some markers of metabolic syndrome (blood glucose, insulin resistance, and triglycerides) after consumption of green/roasted coffee blend only in hypercholesterolemic subjects [129]. However, decaffeinated coffee was not used as a control in any of these studies to determine the effect of other phytochemicals in coffee, which includes high amounts of phenolic acids but also caffeine, trigonelline and diterpenes [130]. When the effect of both regular and decaffeinated espresso was studied on the elastic properties of the aorta and wave reflections, a stronger response was observed among non-habitual coffee drinkers compared to habitual drinkers after the intake of both types of coffee [131]. No difference was seen after receiving caffeine alone, suggesting that the phenolic acids in coffee may be responsible for the interindividual variability in the responses, although the effects of other phytochemicals cannot be excluded. Further evidence of the role of coffee phenolic acids was obtained by Jokura et al., who gave coffee polyphenol extract, consisting mostly of chlorogenic acids, to 19 male volunteers [132]. A significantly increased level of GLP-1, involved in anti-diabetic and beneficial cardiovascular effects, was observed only in individuals with a lower insulinogenic index. However, only the levels of chlorogenic acids were measured from the extract, and thus the contribution of other phytochemicals (except caffeine) to the effect cannot be ruled out.

In conclusion, although HCAs have been linked with promising effects associated to human health promotion and disease prevention, the current evidence of their role is far from being robust and conclusive, and more research is needed. The variability in the individual health responses to phenolic acids seems to be related to the metabolic status, gender, dietary habits, and genetic polymorphisms, but the contribution of other dietary bioactives cannot be dismissed. Further studies should be designed, taking into account the potential confounding effects of other plant phytochemicals and their metabolites present in concomitance with HCAs, and the heterogeneity in the responsiveness to HCA consumption should be more carefully investigated.

## Which factors are relevant in the interindividual variation?

**Fig. 2:**
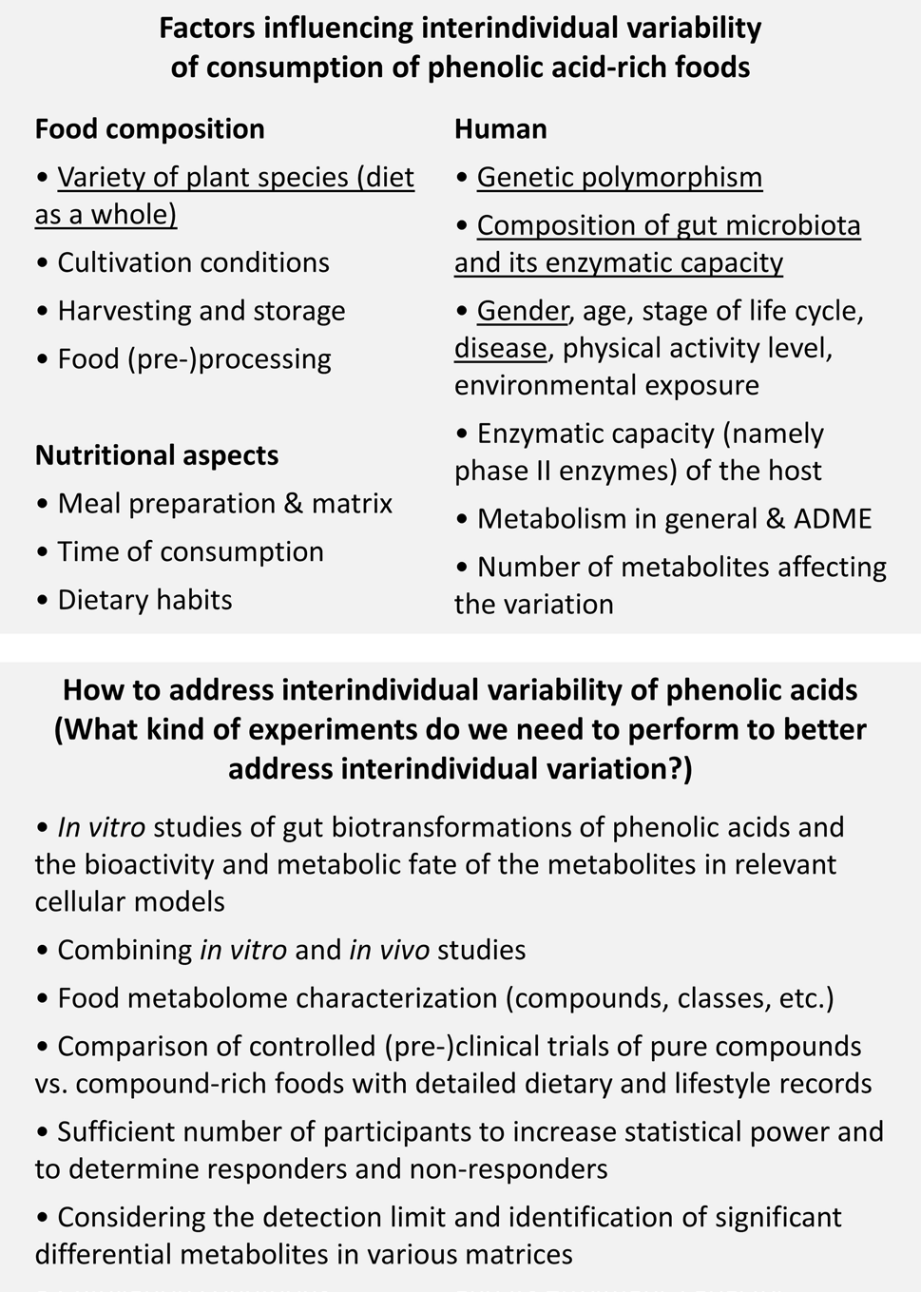
Factors influencing the interindividual variability related to consumption of phenolic acid-rich foods and recommendations for future research. The factors with major impact according to current knowledge are underlined

The scenario is complex when attempting to characterise the large interindividual variability in the metabolism and response of food phytochemicals, such as HCAs, HBAs  and polyphenols sharing partially the same microbial metabolites. Various factors are relevant: controlling the diet of the intervention (contents of the phytochemicals in beverages or food matrices, effects of processing on their release), gut microbial ecology and genetics, and enzymatic plasticity of the host (phase I and II metabolic enzymes, and phase III transporters) and those of the microbiome. Furthermore, beyond the bioactive phytochemical component at study, dietary habits, lifestyle, geographical and other environmental factors influence the biological effect on health and disease state (Fig. 2).

It is clear that free-living people do not eat only single foods, and they seldom obey a controlled diet for a long period of time. However, phenolic acids may contribute to the dietary intake of polyphenols to a substantial degree. As shown in Table 1, the phenolic acid content of polyphenol-rich diet represent almost half of the total polyphenol intake. The metabolic routes of HCAs have been recently elucidated to some extent, and the effect of food processing on the HCAs may cause variation [51, 63–66]. Still, we need to acknowledge that processing, affecting indirectly the bioavailability of phenolic acids, has a limited impact on the interindividual variability, if we consider the whole dietary consumption.

Human interventions, sometimes including a low number of subjects (Table 1), have observed biomarkers of phenolic acid intake or correlations between the intake and health benefits. The subtle differences are discussed with regard to other confounding factors or components. The observed correlations do not represent causality, and thus a better understanding of the gut–host interplay and microbiome biochemistry is becoming highly relevant. The reversible interaction between the diet, the host and the microbiome, and their consequent synergistic effects on health is a challenge to tackle. Because of this three-way interplay, it is important to understand which microbial species correlate with the phenolic metabolites produced at individual level. Microbiological studies, and especially those dealing with ecological communities of microbiomes, including genome-scale models, studying the functional genome of the microbiome, could explain partly interindividual variation of the microbial metabolites [133].

**Fig. 3:**
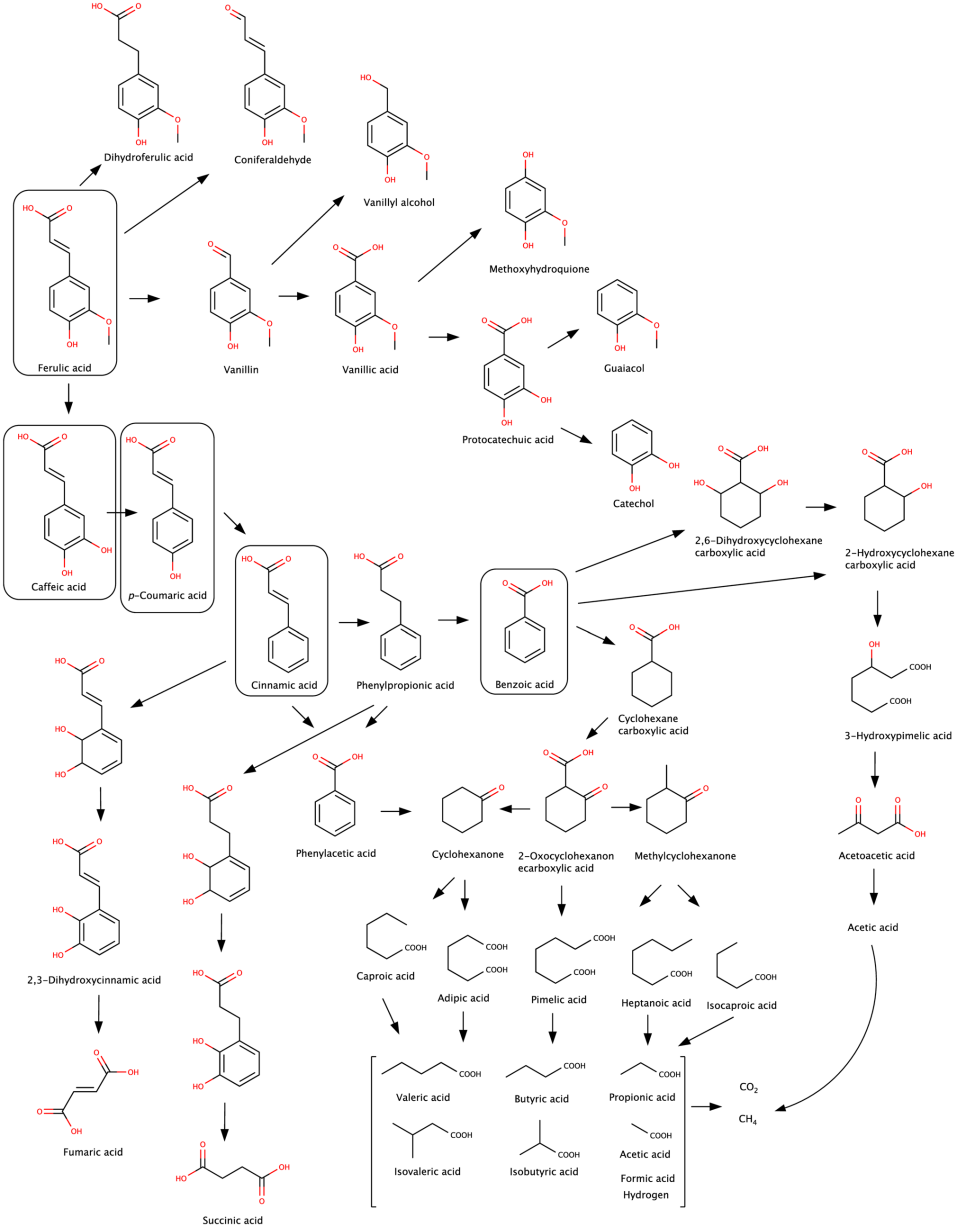
Major pathways of the microbial anaerobic degradation of ferulic acid, caffeic acid and *p*-coumaric acid. The pathways are connected to hydroxybenzoic acids and amino acids

Do we understand the microbial metabolism to a full degree? Microbial transformations of ferulic acid have been described in various environments beyond the human gut microbiota, with concurrent intermediates to those found in faecal matter [134]: oxidation or reduction of the side chains, leading to coniferaldehyde and vanillyl alcohol or dihydroferulic acid; cleavage of the side chain, forming vanillin and eventually vanillic acid via the oxidation of the aldehyde group; and finally, the formation of guaiacol and protocatechuic acid as common intermediates (Fig. 3). Benzoic acid (and benzoyl-CoA, which is readily formed by benzoate CoA ligase) is a kernel intermediate from the anaerobic degradation of aromatic compounds [78, 135]. Therefore, benzoic acid can also be a general degradation product from aromatic compounds, such as vanillin, *p*-cresol, phenol, aniline, aromatic amino acids such as phenylalanine, and polyphenol flavonoids. Benzoic acid, the simplest of all aromatic acids, can be further de-aromatized (partially or completely), forming cyclohexane carboxylic acid and cyclohexanone. Hydroxylated forms of cyclohexane carboxylic acid have also been detected from anaerobic degradation of aromatic compounds. The anaerobic metabolism culminates in ring opening and aliphatic compounds such as caproic acid, adipic acid, pimelic acid, and their hydroxylated forms can be formed [77, 136, 137]. Ultimately, short-chain fatty acids (valeric acid, butyric acid, propionic acid, and acetic acid, and their respective CoA conjugates) accumulate [79]. Nevertheless, despite this key information, the impact of anaerobic microbial metabolism of phenolic acids on interindividual variability is limited and poorly known.

The microbial and endogenous metabolism of phenolic acids has proven to be complex and interlinked, and future studies may reveal additional intermediary or end metabolites, which may have synergistic effects on host. The increased number of bioinformatic tools, including mapping and prediction of biochemical pathways with databases, such as the PhenolExplorer [138], the Atlas of Biochemistry [139], and the biocatalysis/biodegradation database with curated information on environmental degradation of pure compounds [79], could help in the identification of the various still unknown substances. Those compounds may be difficult to connect with phytochemicals because their origin may be challenging to be distinguished from dietary fibre carbohydrates, proteins, and amino acid degradation, which exceed 100–1000-fold the contribution of any non-nutrient phytochemicals in the diet. Therefore, it is worthwhile to focus on the major metabolites that can be identified from hepatic or colonic conversions using in vitro models, and those that can be detected from the body fluids, e.g., plasma and urine. It is worth of a note that the gut microbial metabolism also reduces the chemical complexity of the metabolites, as larger structures, such as polyphenols, are degraded into smaller, simpler structures of microbial phenolic acids, which are the same as those formed from HCAs and benzoic acid derivatives of the diet, as indicated in Table 1. Nevertheless, the large number of metabolites emerging from even one single component enhances the impact of interindividual variation, since the minor metabolites cannot be distinguished as significantly differing components from the control groups.

In vivo and in vitro research should go hand in hand to understand the efficacy of bioactive compounds, and mechanisms involved, to assess health benefits and define recommendations. The pool of circulating metabolites could be related to shorter (hepatic metabolites) or longer (microbial metabolites) residence times and a synergy of these metabolites may cause the effect on health. Regarding not only interindividual variability but also intra individual variability, more research is warranted on gene variants showing how variability in enzymes of liver and the functionality of colonic microbial community affect the levels of phenolic acid metabolites. The variability in the genes and enzymatic activities of the complex system of xenobiotic metabolism is also a large contributor to how an individual handles phenolic acids and the metabolic fate of the compounds. Here, research on drugs proves to be crucial to characterise enzymes and identify genetic polymorphisms, affecting the ability of the host to convert the circulating metabolites also from the diet. Therefore, scientific communities of drug and food research could collaborate to learn more from each other and potential synergistic effects of the diet and drugs on the host metabolism.

Conventionally, human interventions studying health benefits and those studying the metabolite profiles after consumption of phenolic acid-rich foods have been performed separately. Furthermore, the studies are objected to a limited number of food items, and with limited number of subjects, which results in subtle differences between study groups. Only seldom do studies combine the two approaches and approach the whole recommendable diet [85, 114]. It is also a fact that results from intervention studies are only presented as averages and standard deviations or means and interquartile ranges (IQR; ranges of middle 50%), which exhibit the large variations, but make the results difficult to interpret.

Even a “perfect” study design cannot eliminate or completely tackle individual variation. Would it be possible and acceptable to change the approach to handle the data? The responders and non-responders of the observed health effect could be separated, and the affecting factors could be studied analysing the biomarkers of intake (including the host-derived and microbial metabolites) and expression of genes. On the other hand, genetic polymorphism could be the starting point of a study, followed by investigating the metabolism and health responses in a synergistic manner. Thus, health responses could be cross-referenced with metabolite profiles, gene variants, diet, exercise, or other lifestyle factors. This has been used in medical research in case–control studies. Could we adopt new approaches of designing experiments or handling the data to help overcome the interindividual variation? Could artificial intelligence and collection of big data help in identifying critical factors? There is a need for a thorough discussion on how the limiting factor, interindividual variability, could be turned into an asset in an ethical way to better understand and utilize personalized nutrition and the impact of the complete recommended diet on health.

## Abbreviations

ARE: Antioxidant-response element
BMI: Body mass index
FMD: Flow-mediated dilation
GIP: Glucose-dependent insulinotropic polypeptide
GLP-1: Glucagon-like peptide-1
HBA: Hydroxybenzoic acids
HCA: Hydroxycinnamic acids
LAB: Lactic acid bacteria
PAD: Phenolic acid decarboxylase
